# *Candida auris* fungaemia outbreak in a tertiary care academic hospital and emergence of a pan-echinocandin resistant isolate, Greece, 2021 to 2023

**DOI:** 10.2807/1560-7917.ES.2024.29.45.2400128

**Published:** 2024-11-07

**Authors:** Joseph Meletiadis, Maria Siopi, Bram Spruijtenburg, Panagiota-Christina Georgiou, Maria Kostoula, Sophia Vourli, Frantzeska Frantzeskaki, Elisabeth Paramythiotou, Jacques F Meis, Iraklis Tsangaris, Spyros Pournaras

**Affiliations:** 1 Clinical Microbiology Laboratory, “Attikon” University General Hospital, Medical School, National and Kapodistrian University of Athens, Athens, Greece; 2 Canisius-Wilhelmina Hospital (CWZ)/Dicoon, Nijmegen, The Netherlands; 3 Radboudumc-CWZ Center of Expertise for Mycology, Nijmegen, The Netherlands; 4 Infection Control Committee, “Attikon” University General Hospital, Medical School, National and Kapodistrian University of Athens, Athens, Greece; 5 Institute of Biosciences and Applications, National Centre for Scientific Research "Demokritos", Aghia Paraskevi, Greece; 6 Second Critical Care Department, “Attikon” University General Hospital, Medical School, National and Kapodistrian University of Athens, Athens, Greece; 7 Department of Critical Care, Laikon Hospital, 11527 Athens, Greece; 8 Institute of Translational Research, Cologne Excellence Cluster on Cellular Stress Responses in Aging-Associated Diseases (CECAD) and Excellence Center for Medical Mycology (ECMM), University of Cologne, Cologne, Germany

**Keywords:** candidaemia, *Candida auris*, outbreak, echinocandin-resistance, Greece

## Abstract

After the start of the COVID-19 pandemic, a rapid rise in reported numbers and wide geographic spread of *Candida auris*-related invasive infections has been observed globally. However, the contemporary epidemiology of *C. auris* fungaemias in Greece remains unknown. An outbreak of *C. auris* bloodstream infections has been ongoing for almost 3 years in a Greek tertiary care academic hospital, with 89 *C. auris*-driven episodes appearing in five waves every 6–7 months following peaks in colonisation rates by 3–4 months. All isolates clustered in clade I and were genetically related, 84% were fluconazole-resistant and all were non-resistant to amphotericin B and echinocandins, except one pan-echinocandin-resistant isolate (*FKS1^S639F^* mutant) recovered from a patient on empiric therapy with anidulafungin. Notably, *C. auris* was in 2023 the most prevalent (34%) cause of candidaemia in our hospital. The accelerated and long-term transmission dynamics of *C. auris* fungaemia underscore the need for rigorous infection control measures, while antifungal stewardship is warranted to contain the selection of echinocandin-resistant isolates.

**Key public health message**

**What did you want to address in this study?**

We wanted to investigate the evolution of a *Candida auris* outbreak ongoing for almost 3 years in a Greek hospital and the factors that contributed to its spread and led to the emergence of *C. auris* as a prominent cause of bloodstream infections.

**What have we learnt from this study?**

Strict adherence to infection prevention measures, particularly at the beginning of an outbreak, is of paramount importance. Any break in these measures can lead to an uncontrolled outbreak. Screening on admission could help to promptly detect colonised patients and isolate them. Without necessary resources (space, personnel, sensitive laboratory techniques), outbreaks are difficult to control.

**What are the implications of your findings for public health?**

The accelerated and long-term transmission dynamics of *C. auris* have caused hospital outbreaks and increased invasive infections in the wake of the COVID-19 pandemic, advocating ongoing vigilance, reliable and timely testing for infections resistant to antifungal treatment, and strict adherence to infection control practices.

## Background

The growing number and worldwide spread of *Candida auris*-related invasive infections pose a challenge in healthcare settings [1,2]. It is well established that *C. auris* has the capacity to efficiently colonise cutaneous surfaces and to reside in hospital niches for an extended period of time, allowing it to spread easily. Infection control breaches, inappropriate use of personal protection equipment, poor adherence to hand hygiene, lapses in standards of care for maintenance of invasive devices and inadequate disinfection of shared medical devices or equipment, favour patient-to-patient as well as inter-facility *C. auris* transmission [3]. It is noteworthy that *C. auris* outbreaks are difficult to control, although there have been instances where transmission of *C. auris* was contained with infection prevention and control (IPC) measures after the occurrence of only a few cases [4-7]. The situation may have been accentuated by the COVID-19 pandemic as the focus of the IPC teams shifted from the reduction of healthcare-associated infections to helping healthcare personnel to safely care for COVID-19 patients and mitigate the in-hospital spread of this viral infection [3].

Remarkably, *C. auris* cases were recorded during the pandemic in countries across five continents that had never reported this species before the COVID-19 pandemic, namely Brazil [8], Guatemala [8], Mexico [8], Peru [8], Denmark [4], Portugal [9], Romania [10], Nigeria [11], Jordan [12], Lebanon [13] and New Zealand [14]. Greek hospitals had not been confronted with *C. auris* outbreaks before the pandemic [2]. *Candida auris* bloodstream infections (BSIs) have been described in Greece mainly in COVID-19 intensive care unit (ICU) patients [15,16].

## Outbreak detection

In March 2021, a patient (Patient 1) was admitted to Attikon University hospital (AUH, Athens, Greece) for reasons unrelated to COVID-19. The patient had no history of travel abroad before admission but a recent (within 1 month) hospitalisation at another Greek hospital in our region. On day 5, the blood culture (BC) taken on admission revealed *C. auris*. After the notification of a *C. auris* positive BC, this index patient was screened for *C. auris* (axilla and groin skin swabs) and found to be colonised. A retrospective database search showed that *C. auris* had not been previously identified in our centre. The patient was treated in contact isolation and micafungin (100 mg every 24 h) was administered for 14 days. Subsequent BCs (days 7 and 16) were negative for *Candida,* and the patient was discharged on day 28. Environmental cleaning with quaternary ammonium followed by 5,000 ppm chlorine and steam was implemented on the wards where the patient was treated, while 1,000 ppm chlorine-based disinfectants were used to clean other rooms. Screening of patients who were hospitalised in the same room during the same period or occupied the same bed immediately after were negative for *C. auris*, as was weekly environmental sampling up to 2 weeks after discharge at different time intervals.

In June 2021, a patient (Patient 2) was admitted to a COVID-19 ward in our hospital. On day 1 of hospitalisation, *C. auris* was isolated from bronchial secretions of another patient who had previously occupied the same bed. Therefore, screening for *C. auris* was conducted in all patients sharing the room as well as in high-touch surfaces (bed surfaces and handles of beds, bedside tables, medicine trolleys, sink basins, light switches and door handles), but no *C. auris* was detected. On day 3, Patient 2 was transferred to the ICU without being screened for *C. auris* on ICU admission. On day 28, he developed *C. auris* BSI. No *C. auris* colonisation screening was performed during his 26-day stay in the ICU. The patient was screened (axilla and groin skin swabs) only after the notification of a *C. auris*-positive BC (day 30) and was found to be colonised with *C. auris*. Treatment with caspofungin (50 mg every 24 h) for 12 days was initiated, and follow-up BCs (days 35 and 46) were negative for *Candida*. On day 52, the patient was discharged from the ICU.

After these two *C. auris* fungaemia cases, a long-lasting and large outbreak of *C. auris* BSIs developed in our hospital. We describe the genotypic data, susceptibility testing results and colonisation data, and report the emergence of a pan-echinocandin resistant isolate after repeated exposure to anidulafungin.

## Methods

### Study setting and definitions

All microbiologically confirmed *C. auris* BSIs in patients hospitalised in AUH, until 31 December 2023 were retrospectively analysed. The AUH is a modern 750-bed teaching hospital that had served as the COVID-19 referral centre of the second regional health authority (western and southern Attica region as well as most Aegean islands) since the beginning of the pandemic. Several changes in its routine workflow have been implemented in order to accommodate the surge of COVID-19 patients. The hospital has progressively returned to the pre-COVID-19 levels of function from late 2022 onwards, attending high complexity cases on adult, paediatric and neonatal ICUs, haematology and oncology wards as well as bone marrow transplantation and HIV/AIDS units.

For this study, we defined candidaemia as the recovery of *Candida* spp. from at least one BC set during hospitalisation. An episode was defined as ICU-acquired candidaemia when signs and symptoms of infection developed more than 48 h after ICU admission. Carriage was defined as the detection of *C. auris* from axilla and groin skin swab samples. Once a patient was found to be *C. auris* positive, no further screening was performed during hospitalisation. Hence, only new colonisation cases were recorded. COVID-19 patients were those that had compatible clinical symptoms and tested positive for severe acute respiratory syndrome coronavirus 2 (SARS-CoV-2) RNA in respiratory specimens using commercial real-time RT-PCR assays. Patients’ demographic data (sex and age), medical unit at the onset of infection, mycological findings and outcome during hospitalisation were obtained from the hospital’s computerised databases.

### Microbiological investigation

Positive BCs were inoculated onto blood agar, MacConkey agar and chocolate agar, incubated at 37 °C, as well as Sabouraud glucose agar with gentamicin and chloramphenicol, and chromogenic Brilliance *Candida* agar incubated at 30 °C, for up to 48 h (all agar plates were purchased from Oxoid). Axillary and inguinal surveillance swabs were suspended in tubes containing 4 mL in-house-prepared, sterile Sabouraud dextrose liquid medium (Oxoid) supplemented with 50 mg/L chloramphenicol (Applichem) and 10% w/v NaCl (Applichem) and were incubated at 42 °C for 48 h. Then, 40 µL of the suspension were inoculated onto Sabouraud glucose agar with gentamicin and chloramphenicol and chromogenic Brilliance *Candida* agar and incubated at 42 °C for 48 h.

Both bloodstream and carriage isolates were identified by MALDI-ToF mass spectrometry (Bruker Daltonics). We assessed the in vitro antifungal susceptibility of the bloodstream isolates with the Clinical and Laboratory Standards Institute (CLSI) M27-Ed4 reference broth microdilution method [17] using the United States Centres for Disease Control and Prevention (CDC)’s tentative breakpoints for resistance to amphotericin B, fluconazole and echinocandins [18] and species-specific CLSI epidemiological cut-off values for the other azoles [19]. We used the recommended *Candida krusei* ATCC 6258 and *Candida parapsilosis* ATCC 22019 as quality control strains. A *C. auris*-specific short tandem repeat (STR)-based assay was used for genotyping of bloodstream and carriage isolates [20]. Any echinocandin-resistant isolates were subjected to *FKS1*-hot spot 1 sequencing as previously described [21].

## Results

**Figure 1:**
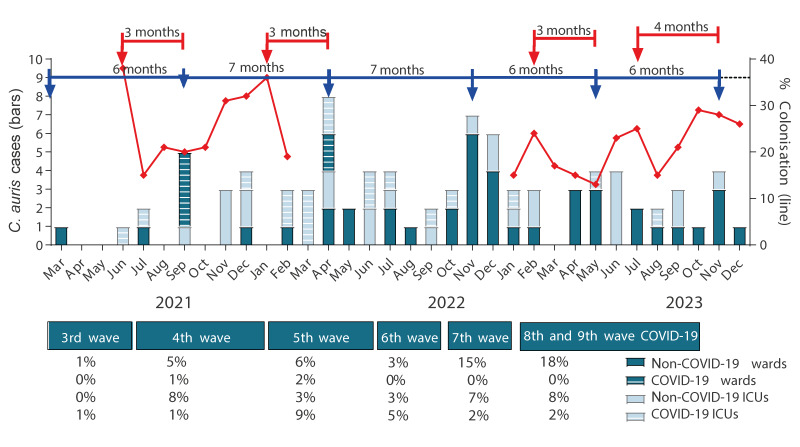
Epidemiological curve of *Candida auris* bloodstream infections during the COVID-19 transmission waves in different patient populations, Greece, 2021–2023 (n = 89)

BSI: bloodstream infection; ICU: intensive care unit.

Two and a half years after the first two BSI cases were identified, the *C. auris* outbreak was still ongoing and intra-hospital transmission was evident with 87 additional BSI episodes recorded until the end of 2023 (14 in 2021, 43 in 2022 and 30 in 2023). The epidemic curve was characterised by elevated case numbers occurring every 6–7 months (September 2021, April 2022, November 2022, May 2023 and November 2023). The median age was 67 years (range: 22–97) and 58 of 89 (65%) were male patients. The episodes occurred in patients with COVID-19 (3% on wards, 20% in ICUs) and without COVID-19 (48% on wards, 29% in ICUs), with a shift from COVID-19 to non-COVID-19 ICUs and wards over time. Almost half of the patients had concurrent multidrug-resistant Gram-negative bacteraemia. Active surveillance performed in 996 ICU patients during the early (May 2021–February 2022; n = 444) and the late (January–December 2023; n = 552 patients) phase of the outbreak, respectively, identified 15–38% and 13–29% of the individuals screened per month colonised with *C. auris,* with an increase of carriage preceding an increase of patients with candidaemia by 3–4 months (Figure 1). The duration of hospital stay until colonisation ranged from 18 to 24 days. For every 5–10% new colonisation cases per month, we found one case of BSI per month. The crude mortality rate during hospital stay was 70% (62 patients, whereof 13 had COVID-19, seven had malignancy and two were patients with malignancy and COVID-19). Notably, *C. auris* was the leading cause of candidaemia in our hospital in 2023 (34%).

The timing of the COVID-19 transmission waves in Greece was as follows: first wave: 26 February–31 May 2020; second wave: 1 June 2020–17 January 2021; third wave: 18 January–21 June 2021; fourth wave: 22 June–19 December 2021; fifth wave: 20 December 2021–5 June 2022; sixth wave: 6 June–18 September 2022; seventh wave: 19 September 2022–19 February 2023; eighth wave: 20 February–19 November 2023; ninth wave: 20 November–27 December 2023 [43].

The red and blue arrows represent the increase in % ICU colonisation and BSI episodes, respectively. The red vectors represent the periods between the colonisation and BSI increase, whereas the blue vectors represent the periods between the BSI increases. The percentages below the COVID-19 waves correspond to the incidence of *C. auris* BSIs (number of episodes/89) in different patient populations.

### Genotypes and antifungal susceptibility patterns

**Figure 2:**
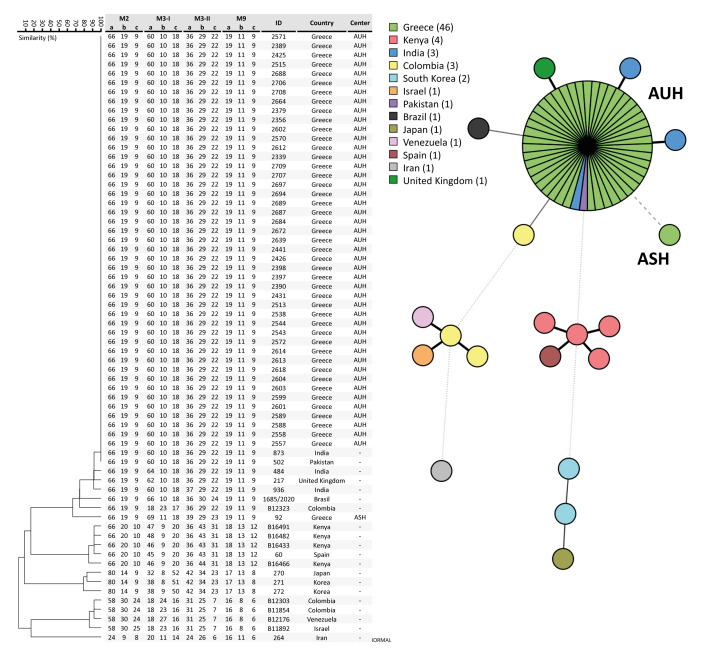
Dendrogram of short tandem repeat genotypes and minimal spanning tree of representative *Candida auris* isolates from bloodstream infection and colonisation, Greece, 2021–2023 (n = 45) plus the first *C. auris* isolated in 2019 in Greece (ID 92)

All BSI and carriage isolates clustered in *C. auris* clade I and had high degree of relatedness (Figure 2). The *in vitro* antifungal susceptibilities were available for 74/89 (83%) bloodstream isolates. All bloodstream isolates were interpreted as amphotericin B-non-resistant, 62 of the 74 were fluconazole-resistant, and all but one were echinocandin-non-resistant. Most isolates were wild-type (WT) to the other azoles [16].

Branch lengths indicate the similarity between isolates with thick solid lines (variation in one allele), thin solid lines (variation in two alleles), thin dashed lines (variation in three alleles) and thin dotted lines (variation in four or more alleles).

The isolates originating from the outbreak in our hospital shared the same genotype. Identical STR data have been previously recorded for isolates in India and Pakistan, while the first documented strain in Greece (ASH, isolate 92_Greece) [28] demonstrated variations in markers M3-Ia, M3-Ib , M3-IIa and M3-IIc [20].

**Figure 3:**
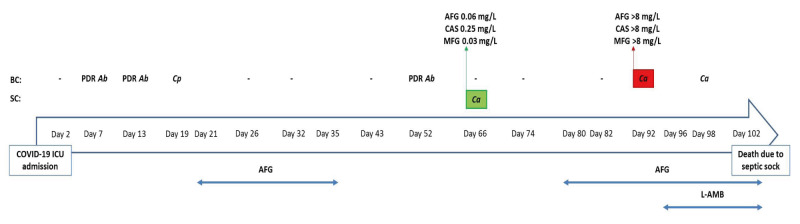
Timeline of microbiological investigation and antifungal therapy of in vivo evolution to *Candida auris* echinocandin resistance in one patient, Greece, March 2022–June 2022

A previously colonised patient with echinocandin-non-resistant *C. auris* developed pan-echinocandin-resistant *C. auris* breakthrough BSI during empiric treatment with anidulafungin with a total exposure of 4 weeks (Figure 3). Sequencing of *FKS1* revealed that the bloodstream isolate was a *FKS1^S639F^* mutant, whereas the carriage isolate had a WT phenotype. The strains had identical STR genotypes. Resistance to echinocandins was not detected in other *C. auris* isolates during the study period and none of the other patients have so far received antifungal treatment.

*Ab*: *Acinetobacter baumannii*; AFG: anidulafungin; BC: blood culture; *Ca*: *C. auris*; CAS: caspofungin; *Cp*: *Candida parapsilosis*; ICU: intensive care unit; L-AMB: liposomal amphotericin B; MFG: micafungin; PDR: pan-drug-resistant; SC: surveillance culture.

Echinocandin-non-resistant and echinocandin-resistant *C. auris* isolates are shaded in green and red, respectively.

## Outbreak control measures

In 2021, the Hellenic National Public Health Organisation issued recommendations for the management of *C. auris* transmission and potential outbreak situations. These were updated in 2023 and included recommendations for detection and identification of *C. auris* in the microbiological laboratory as well as for infection control measures for sporadic cases and during outbreaks, such as patient isolation, contact precautions, screening of patients hospitalised in the same room or ward with a colonised case, adherence to the standard hand hygiene protocols and enhanced environmental cleaning.

Based on the above, the infection control committee of our hospital has issued relevant recommendations on IPC measures in an attempt to reduce or prevent the spread of *C. auris*. Early detection of *C. auris* colonisation is considered the basis for rapidly identifying cases, implementing precautionary measures and preventing further transmission of the pathogen. Selective liquid broth culture followed by subculture on chromogenic media were used in surveillance cultures, and all species were identified to the species level. When *C. auris* was recovered from a diagnostic or screening specimen, the IPC nurses informed the chief nurse and the attending physician of the department where the colonisation/infection was detected. The detection of *C. auris* triggered immediate screening of all current ward mates, high risk patients for colonisation and all patients admitted to and discharged from ICU. Axilla and groin of non-colonised patients were screened weekly, while colonised patients were not screened again as they were considered colonised for the entire duration of hospitalisation. A colonised or infected patient was placed in a single room, where this was possible, with dedicated equipment and nursing staff. If single room isolation with dedicated equipment and nursing staff were not possible, patients with *C. auris* were placed in cohorts, shared equipment and high-touch surfaces were thoroughly cleaned and disinfected, and medical care of patients colonised or infected with *C. auris* was provided at the end of the shift. Patients took baths with chlorhexidine particularly before discharge. Environmental cleaning was compulsory in areas occupied by colonised or infected patients using high-strength (5,000 ppm) chlorine-based disinfectants, and all clothing including lab coats were washed at high temperature. Staff was advised to strictly follow the personal measures of protection. The IPC nurses monitored and reinforced healthcare providers’ adherence with hand hygiene through regular visits and instructions in rooms with colonised patients.

Several factors could have promoted the outbreak’s evolution. During the COVID-19 pandemic, extended wear and reuse of gloves for the care of multiple patients and staff movements across different hospital units was noticed. In addition, AUH received patient transfers from institutions located in the Attica region where *C. auris* cases (carriage and infection) had been documented [22-25]. However, *C. auris* infections are not mandatorily notifiable in Greece, and *C. auris* screening on admission or discharge of patients at risk is not common practice in Greek hospitals. The latter strategy was fine-tuned in our centre from the moment the first *C. auris* BSI was detected. It was applied either on admission or randomly during hospitalisation only for ICU patients, although this was not applied to all ICU patients due to limited resources. When patients need to be transferred between different healthcare facilities, the receiving facility is responsible for implementing *C. auris* IPC measures. Thus, the receiving facility was only notified of a positive or a recent negative result if the patient had been screened for *C. auris* during his/her stay in our hospital. Moreover, when the number of patients colonised/infected with *C. auris* increased substantially, isolation or cohorting until discharge could not be implemented due to space and nursing staff shortages. Contact isolation was feasible at low colonisation rates <5%, whereas for at higher colonisation rates (most of the times), cohorting strategies were implemented. Lastly, we cannot rule out persistent environmental niches of the pathogen or patient-to-patient transmission since environmental sampling was only performed in response to the identification of the first two BSIs. However, 5,000 ppm chlorine was used instead of standard 1,000 ppm as the high concentration of chlorine was more efficient to kill *C. auris* [26,27]. Because no positive environmental samples were found, we suspect that the main route of transmission was the healthcare workers’ hands. Stopping patient admittance was not possible, and staff was not screened. Control measures may have had some effect as colonisation rates were low in some months during the outbreak but lack of strict and continuous implementation of control measures affected the course of the outbreak.

## Discussion

We have described the 3-year period March 2021 to December 2023 of a still ongoing outbreak of *C. auris* fungaemia in the Greek tertiary care hospital AUH. There were 89 *C. auris*-driven episodes, appearing in five waves every 6–7 months following increased colonisation rates by 3–4 months, with 1 BSI observed for every 5–10% new colonisation cases per month. All isolates clustered in clade I and were genetically highly related, 84% were fluconazole-resistant and all were non-resistant to amphotericin B and echinocandins, except one pan-echinocandin-resistant isolate (*FKS1^S639F^* mutant) recovered from a patient on antifungal therapy with anidulafungin.

After the first report of *C. auris* colonisation in a Greek cystic fibrosis patient in 2019 [28], *C. auris* BSIs have been sporadically recorded until the end of 2023 in COVID-19 ICU patients hospitalised in centres located in Athens [15,22,23]. Interestingly, *C. auris* BSIs were not identified during the pandemic in southern (Crete; COVID-19 patients, from March 2020 to August 2022) [29], southwestern (Patras; ICU patients, from April 2020 to August 2021) [30] and northwestern (Ioannina; general patient population, 2020–2021) [31] Greece. Of note, the European Centre for Disease Prevention and Control has recently incorporated Greece in the countries at critical risk of spread of this pathogen (epidemiological stage 4) as a consequence of multiple outbreaks reported between 2020 and 2021, although without providing details on their classification (infection or carriage), their geographical distribution and the patient populations involved [2]. In all but one case [15], available information relied solely on descriptive data.

Herein we provide a genotype-informed snapshot of the evolution of an ongoing outbreak including colonisation data, which is not restricted to distinct units, revealing its dynamic nature. Notably, there was a prolonged 3-month lag between the first two episodes, as previously described [32], probably due to increased vigilance after the identification of the index case that was later reduced under the pressure of the fourth and at that time largest COVID-19 transmission wave in our country. Interestingly, a periodicity was observed for increases in BSI and colonisation cases, with BSI increases occurring every 6–7 months and 3–4 months after the colonisation increase, at a BSI:colonisation ratio of between one in five and one in 10 per month. However, we performed active screening only in ICUs, while the BSI incidence referred to all hospital wards. Active screening was not performed during the two peaks of BSI incidence, and the last two increases were of small magnitude. We assumed that the ICU environment reflected the burden of colonisation in hospital as patients were transferred from ICU to wards and vice versa. Furthermore, despite the gaps in active screening and the small size of the last two increases, the pattern remained the same throughout most of the study period. *Candida auris* may survive up to a month on surfaces [33,34], whereas patients are decolonised 8.6 months after being discharged to a community setting with no intervention [35]. Although we cannot exclude the environment as a source of the outbreak, the shorter survival time of *C. auris* on surfaces and the effectiveness of disinfection with 5,000 ppm chlorine [26,27] on the one hand, and the longer time required for patients to be decolonised as well as the temporal and quantitative association between BSI and colonisation on the other hand, indicate that colonised patients may have been the main source of the outbreak. It should be noted that its periodic nature corresponded to the COVID-19 transmission waves in our country and could reflect hospitalisation load, as previously described [36].

All isolates had similar antifungal susceptibility profiles, they clustered in clade I, and genomic surveillance traced the outbreak to the clonal expansion of a single lineage, which differed from that of the first *C. auris* isolate described in Greece [28], indicating an independent single introduction to our hospital. However, multiple independent introductions cannot be ruled out as isolates from other hospitals share the same STR genotypes as isolates from AUH (data not shown). Although whole genome sequencing (WGS) might be required to determine the precise relatedness of strains with identical STR genotypes, the microsatellite markers used here demonstrate high discriminatory power when compared to WGS [20,37]. Clinical and environmental *C. auris* isolates isolated from different hospitals within the Attica region also belonged to clade I [25]. During the COVID-19 pandemic, there has been a considerable transfer of patients, particularly from smaller to large hospitals such as ours, which could have contributed to an active dispersion of *C. auris*, as previously reported [38]. Whether widespread clusters have emerged across our region remains unknown. However, as the mean duration of hospital stay until colonisation was 18–24 days, most cases have resulted from intra-hospital transmission.

Most (91%) *C. auris* isolates are resistant to fluconazole, which is in agreement with our findings [39]. Nevertheless, persistent or breakthrough *C. auris* BSIs caused by fluconazole-non-resistant strains (CLSI MICs 2–8 mg/L) have been reported [39], which casts doubt on the CDC’s tentative breakpoint of 32 mg/L [18]. Echinocandins are currently labelled as first-line therapy for *C. auris* invasive infections [40]. Notably, we identified an echinocandin-resistant isolate (*FKS1^S639F^* mutant) that evolved in vivo upon repeated exposure to anidulafungin for 2 weeks as targeted therapy and after 44 days for another 2 weeks as empirical therapy in a colonised patient. Breakthrough *C. auris* infections, mainly catheter-related, associated with *FKS1* mutant strains have been described, indicating their therapy-induced selection [41,42]. Similar to our findings, echinocandin-resistant *C. auris* BSIs have been reported in patients previously tested positive for skin carriage who had experienced prolonged (19–74 days) echinocandin treatment [41].

Among the limitations of the study are that surveillance cultures were performed only in ICU patients and not during the entire period of the outbreak. Once they were found to be positive, colonised patients were not screened again in order to check whether they remain positive during their hospitalisation. The exact number of colonised patients per month is not known, and extensive environmental sampling was not conducted to identify potential niches. Levels of adherence to IPC measures were not monitored over time for wards and ICU separately, and whole genome sequencing of isolates was not performed to verify relatedness to greater extent. 

## Conclusion

The accelerated and long-term transmission dynamics of *C. auris* have emerged in the wake of the COVID-19 pandemic, advocating ongoing vigilance and strict adherence to infection control practices. Most important are screening on admission of all patients who were previously hospitalised or had visited health-care facilities or hospitals in endemic areas (a national monitoring system could help to identify carriers), point prevalence studies to detect colonised patients, high-quality sampling procedures (screen at least three sites, e.g. axilla, groin and nose, with PCR assays and cultures), single room contact isolation or cohorting with dedicated nursing staff and rigorous environmental cleaning of high-touch surfaces, laboratory coats and particularly of reusable equipment. Prompt implementation of surveillance and antifungal stewardship is warranted to contain the selection and spread of echinocandin-resistant *C. auris*.
